# Internal Soft-Tissue Anatomy of Cambrian ‘Orsten’ Arthropods as Revealed by Synchrotron X-Ray Tomographic Microscopy

**DOI:** 10.1371/journal.pone.0042582

**Published:** 2012-08-01

**Authors:** Mats E. Eriksson, Fredrik Terfelt, Rolf Elofsson, Federica Marone

**Affiliations:** 1 Department of Geology, Lund University, Lund, Sweden; 2 Department of Physics, Lund University, Lund, Sweden; 3 Department of Biology, Lund University, Lund, Sweden; 4 Swiss Light Source, Paul Scherrer Institut, Villigen, Switzerland; Ludwig-Maximilians-Universität München, Germany

## Abstract

The world-famous ‘Orsten’ Konservat-Lagerstätte has yielded detailed information about Cambrian arthropods and their morphology. Internal organs or soft tissues have, however, rarely been reported, an obvious palaeobiological drawback. In this study, we employed synchrotron radiation X-ray tomographic microscopy (SRXTM) to study microscopic ‘Orsten’ arthropods from the Cambrian of Sweden: *Skara minuta* and two phosphatocopine species, *Hesslandona* sp. and *Hesslandona trituberculata*. This exceptionally high-resolution technique reveals internal organs or soft tissues that allow detailed comparison with equivalent structures in extant crustaceans and functional inferences to be made. The *S. minuta* specimen shows the digestive system and muscles that extend to the extremities. The slanting anterior portion of the head and anterior position of the mouth with a straight oesophagus suggest a primarily brushing and scraping way of feeding. The prominent head appendage muscles indicate muscle strength and good capacity for food manipulation. In the phosphatocopines the bulbous labrum is one of the most prominent morphological structures of the body. All specimens analysed reveal pairs of muscle bundles within the labrum. Based on comparisons with extant crustacean relatives, these muscles would fulfil the function of moving the labrum up and down in order to open the buccal cavity. The results of this pilot study demonstrate that there is still much to be learned about the ‘Orsten’ taxa.

## Introduction

The ‘Orsten’ Lagerstätte from Kinnekulle, on the southern border of Lake Vänern, Sweden, contains remarkably well-preserved minute fossils from bituminous limestones (‘Orsten’) of uppermost mid-Cambrian through Furongian (upper Cambrian) age (e.g. [1]–[6]). The discovery of these remarkable fossils in the mid-1970s has been followed by a sequence of investigations revealing, among other things, morphological details of exceptional interest for understanding the evolution of, and relationships among, early arthropods. The famous ‘Orsten’ taxa have provided significant insights into the Cambrian biota and early Phanerozoic metazoan evolution.

The ‘Orsten’ metazoans are represented by ecdysozoans (moulting animals), including the scalidophoran nemathelminths and arthropods, all in the size range of 2 mm or less (e.g. [3]–[5], [7]). Most of the fossils are arthropods; they include lobopodians, tardigrades, pentastomids, chelicerates, agnostoids, phosphatocopines and skaracarids [4].

By contrast to the phosphatised ‘Orsten’ taxa, the conventional record of shelly fossils in the uppermost mid-Cambrian through Furongian of Sweden is dominated by agnostoids and polymerid (in particular olenid) trilobites (e.g. [8]–[11]), commonly occurring in great abundance in shales and limestones. Although approximately one hundred fairly well-preserved, juvenile specimens of the agnostoid *Agnostus pisiformis* have been recovered in ‘Orsten’-type preservation [2], only one specimen interpreted as a polymerid trilobite hypostome with associated soft tissues has hitherto been discovered [12]. This collectively suggests that the arthropod faunas of this age are taphonomically biased and that the dominance of polymerids and agnostoids in the conventional fossil record does not necessarily represent the true, original faunal composition of arthropods.

The external morphology of the ‘Orsten’ species has been thoroughly described (e.g. [1]–[7] and references therein). However, the internal organs and tissues (such as intestines and muscles) of these fossils have rarely been addressed [4]. Müller and Walossek ([13]:pl. 1, fig. 8) noted that a preserved ‘steinkern’ of *Skara* might represent the gut, an assumption that is confirmed in the present investigation. Moreover, Maas *et al*. ([4]:fig. 4E,F) noted the preservation of guts, which they observed in skaracarids with a cracked-open cuticula. In the same paper, Maas *et al*. documented a pentastomid arthropod with exposed muscle strands in the head region ([4]:fig. 4G), which they referred to as the only known example of unequivocally internal matter in ‘Orsten’-type preservation.

The limited knowledge of internal anatomy is a tantalizing drawback of the ‘Orsten’ fossils and limits the extent to which palaeobiological conclusions can be drawn. The present investigation provides, by using synchrotron radiation X-ray tomographic microscopy (SRXTM), a chance to at least partly overcome that problem. Our use of SRXTM and 3D-rendering techniques applied to specimens of well known ‘Orsten’ taxa from the Cambrian of Sweden has enabled internal structures to be revealed, a task that would be difficult with conventional techniques such as scanning electron microscopy (SEM). Moreover, being a non-invasive technique, SRXTM allows for internal structures of unique fossils to be studied without destroying the specimens. Our work therefore provides a promising approach to understand the internal anatomy and functional morphology of these exceptionally well-preserved microscopic animals.

## Materials and Methods

### Geological setting and sample locality

Mount Kinnekulle is an erosional outlier in the province of Västergötland, south-central Sweden, comprising Cambrian to Silurian strata capped by dolerite intruded as sills during Carboniferous through Permian times [14], [15]. The uppermost middle Cambrian through Furongian (uppermost Cambrian) strata of Mount Kinnekulle crop out in a few natural exposures and a number of abandoned alum shale quarries (e.g. [3]:fig. 2; [8]:fig. 18; [16]:fig. 2). These strata consist of interfingering beds and layers of black alum shale and bituminous limestone (colloquially referred to as ‘stinkstone’ or ‘Orsten’). Agnostoids and polymerid trilobites, predominantly olenids, occur frequently in the succession. Biostratigraphically, the exposed succession spans the *Lejopyge laevigata* Zone through the *Peltura lobata* Zone of the Alum Shale Formation [17]; however, several stratigraphic gaps occur within the succession [14].

The material reported herein was collected from the ‘Transformatorstationen’ locality at Blomberg, on the southwestern part of Mount Kinnekulle (N58°32.558′; E13°19.910′). This locality exposes less than 2 m of bituminous limestones with a few, thin alum shale beds. All ‘Orsten’ samples were collected from the lowermost part of the exposure and belong to the *Agnostus pisiformis* Zone (Guzhangian Stage, or uppermost mid-Cambrian). The required permits for the described field studies were obtained from the land owner.

### Sample digestion, picking and material

In the search for phosphatised ‘Orsten’ fossils we followed the results of Maeda *et al*. [6] and targeted coprolite-rich beds. Slabs of such lithology, weighing approximately 0.5–4 kg each, were digested in pH-monitored buffered acetic acid, following the techniques described by Jeppsson *et al*. [18]. The pH was adjusted to >3.6 in order to avoid corrosion of phosphatic fossils. After digestion the resulting residue was rinsed through a 63 µm sieve cloth. Subsequently, the residue was gently washed into a glass vial with deionized water in order to prevent growth of mould and algae. The residue was carefully investigated for exceptionally preserved microfossils under a binocular light microscope. Specimens of interest were handpicked using a fine brush and stored submerged in water, to avoid damage prior to analyses.

The ’Orsten’ arthropods analysed and discussed herein include one specimen (the only one recovered from our sample residues) of *Skara minuta*, two phosphatocopines assigned to *Hesslandona* sp., and one phosphatocopine assigned to *Hesslandona trituberculata* (see [3]). Approximately 15 phosphatocopines with ventral body details were recovered from our residues. For this pilot study, the three most complete and well-preserved specimens were selected for analysis, in order to increase the chance of finding internal soft-tissue structures.

All figured ‘Orsten’ specimens are stored at the Department of Geology, Lund University, Lund, Sweden, with repository number LO (for Lund Original).

### Electron microscopy

The transmission electron microscope (TEM) micrographs of the extant mystacocarid *Derocheilocaris typica* were prepared at the Department of Biology, Lund University, Sweden, using the procedure described in detail by Elofsson and Hessler [19]. The scanning electron microscope (SEM) methods, including fixation techniques, and set-up for the same extant crustaceans were described by Elofsson and Hessler [20]. Complementary SEM studies of fossil specimens were performed using a Hitachi S-3400N instrument at the Department of Geology, Lund University, Sweden.

### Synchrotron methods and settings

**Figure 1:**
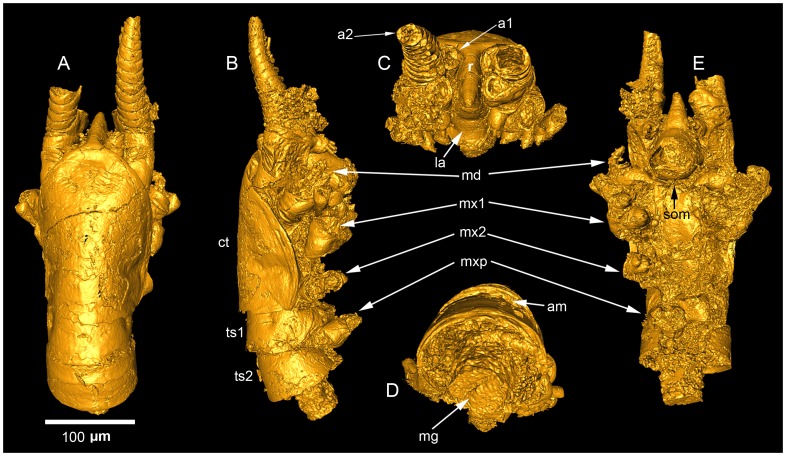
3D-rendering (isosurface) of a SRXTM dataset of *Skara minuta* (LO 11408 t). (A) dorsal, (B) lateral, (C) anterior, (D) posterior, and (E) ventral views. Abbreviations: a1, first antenna; a2, second antenna; am, arthrodial membrane; ct, cephalothorax; la, labrum; md, mandible; mx1, first maxilla; mx2, second maxilla; mxp, maxilliped; r, rostrum; som, site of mouth; ts1, first thoracic segment; ts2, second thoracic segment.

The fossil specimens were analysed using synchrotron radiation X-ray tomographic microscopy (SRXTM) at the TOMCAT beamline of the Swiss Light Source, Paul Scherrer Institute, Switzerland [21]. Some of the specimens were mounted on double-sided adhesive tape on a sample stub and analysed (see below). Other specimens were stacked vertically in thin-walled, low X-ray scattering, capillary glass tubes with an outer diameter of 500 µm, a wall thickness of 10 µm, and a height of 80 mm. The base of the capillary tube was first filled with 10–15 glass beads with a diameter of 25–30 µm. The fossil specimens were placed on top of this stack of beads, each specimen also separated by one bead, which enabled each specimen to be separately scanned. The capillaries were subsequently mounted on sample holders using melted bees' wax and the uppermost, empty part of the capillaries were broken off (see a similar set-up in [22]:fig. 1). In order to optimise the contrast, the beam energy was set to 12 keV. The X-ray radiation transmitted by the sample was converted into visible light by a 20 µm thick Ce-doped LuAG scintillator screen (Crytur, Turnov, Czech Republic). Projection images were magnified by microscope optics and digitised by a high-resolution CCD camera with a 2048×2048 pixel chip and a pitch of 7.4 µm (PCO2000; PCO GmbH, Kelheim, Germany). The optical magnification was set to 20×, resulting in cubic voxels of 0.37 µm in the reconstructed data sets. For each scan, 1501 projections were acquired along with dark and flat field images. The exposure time was 200 ms for each projection, thus the complete data set was acquired in approximately 15 min. The tomographic reconstructions were performed on a 60-node Linux PC cluster using a highly optimised routine based on the Fourier transform method and a gridding procedure [23]. The resulting tif micro-tomograms, or slices, were imported and rendered, using the Voxler2 software package, into 3D-images that could be studied from every angle and virtually cut into different planes.

## Results

Diagenetic phosphatisation can produce a variety of shapes and textures that may be mistaken for fossilised soft tissues [24]–[26]. Herein, criteria such as symmetry, position within the body cavity, continuity and, perhaps most importantly, comparisons with equivalent structures in extant crustaceans with a similar degree of development, have formed the basis for our assessments and allowed structures to be distinguished from taphonomic and/or diagenetic artefacts (cf. [4], [13]).

### *Skara minuta*

The *Skara minuta* specimen (LO 11408t) measures 400 µm in length (note, however, that the posterior portion is lacking) and 130 µm in width and is preserved in minute detail (Fig. 1A–E). In addition to the cephalothorax and the first two thoracic segments, the specimen includes the proximal portions of the first and second antennae, mandibles, first and second maxillae, and maxillipeds (Fig. 1B, C, E). For a detailed description of *S. minuta*, as well as the other known skaracarid species from Sweden, *Skara anulata*, see Müller and Walossek [13]. Herein, we focus on the preserved internal tissues.

**Figure 2:**
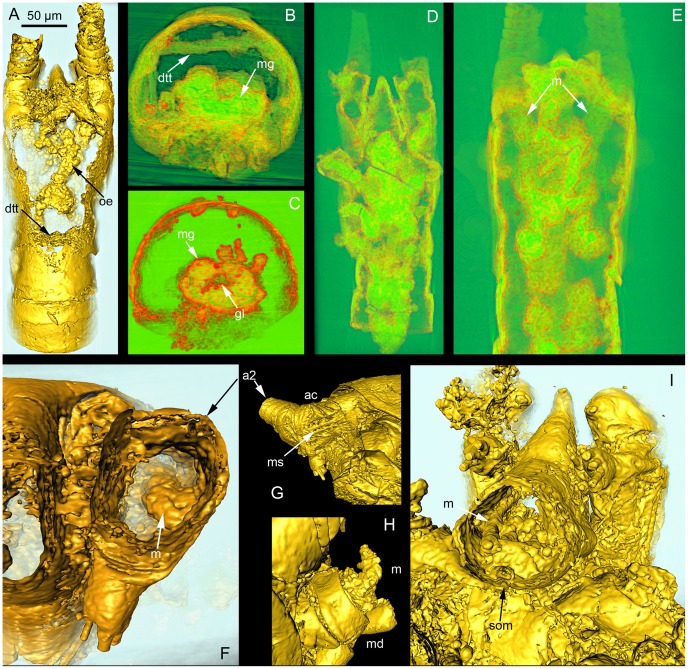
3D-rendering of a SRXTM dataset of *Skara minuta* (LO 11408 t). (A) Ventral view (cropped isosurface) showing the oesophagus, parts of the midgut and a dorsal transverse tendon. (B) Transverse cross-section (cropped volrender) showing the midgut and dorsal transverse tendon. (C) Transverse cross-section (cropped volrender) showing the midgut and gut lumen. (D) Ventral view (volrender) showing the internal plate and muscles extending into the appendages. (E) Dorsal view (volrender) showing the prominent second antennal muscles. (F) Anterior view (cropped isosurface) showing the second antennal muscle and its insertion on the inner wall of the antennal coxa. (G) Isosurface in outer lateral view of the second antennal coxa showing the corresponding muscle scar. (H) Isosurface of right mandible with its protruding muscle exposed. (I) Cropped isosurface in ventral view of the labrum showing the prominent labral muscle and the approximate site of the mouth. Abbreviations: a2, second antenna; ac, antennal coxa; dtt, dorsal transverse tendons; gl, gut lumen; m, muscle; md, mandible; mg, midgut; ms, muscle scar; oe, oesophagus; som, site of mouth.

**Figure 3:**
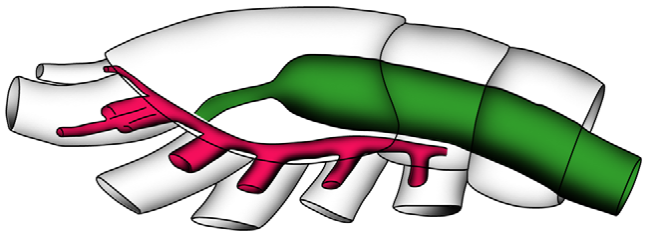
Schematic reconstruction of *Skara minuta* (LO 11408 t). Interpretation of the general architecture of the digestive (green) and muscular systems (red).

The central space of the animal contains structures interpreted as the digestive system, which is composed of the oesophagus (or foregut) and the midgut (Figs. 1D, 2A–C). The oesophagus is a uniform structure approximately 100 µm long and 20 µm in diameter. It has a fairly straight course from the mouth to the midgut due to the forwardly positioned and ventrally directed mouth (Figs. 1E, 2A). The short transition zone from oesophagus to midgut is indistinct (collapsed) in our specimen and we cannot establish whether it is an oesophagus telescoping into the midgut (see below) or a widened portion of the posterior oesophagus.Therefore, the simplified schematic drawing (Fig. 3) merely shows the transition zone as a simple tube. On the other hand, the midgut appears rather well preserved in the animal and distinct from the oesophagus, as judged also by the size and similarity to those structures in extant crustaceans. The midgut is approximately 40 µm in diameter and the irregular lumen is clearly visible in cross-section (Fig. 2B, C). Midgut glands are not observed in our specimen.

Ventrally within the cephalothorax, below the digestive system, tissues from the antennae, mandibles and the two maxillae connect to form a plate, which follows the slanting contour of the ventral face of the animal (Fig. 2A, D, E). These tissues are here interpreted as phosphatised muscles and endoskeleton. The muscles of the thin first antenna leading to the plate are somewhat indistinct. By contrast, the muscles to each of the second antennae are prominent and fan out from the antero-lateral rim of the plate and enter the hollow entrance to the second antennae (Fig. 2A, E). One of the second antennal muscles can be followed to its insertion on the inner wall of the antennal coxa (Fig. 2F). The corresponding muscle scar on the outside is clearly visible (Fig. 2G). Further posteriorly, muscles to the mandibles and maxillae project from the plate to these appendages (Fig. 2D, H). The endoskeleton, serving as the internal insertion of the muscle tissue, cannot be separated from the muscles in our specimen. They are, however, not formed as cuticular ingrowths.

There is no doubt that longitudinal muscles span the length of the thorax and abdomen both ventrally and dorsally in fossil crustaceans, as well as in living relatives [27]. These muscles insert on tendons on the segmental borders. In the analysed specimen of *S. minuta*, c. 15 µm thick rod-like structures occur in the dorsal part of the body cavity below the arthrodial membranes. They follow the outline of, and appear to be associated with, the segment borders (Fig. 2A, B).

Within the labrum, along the inner walls, we observed c. 8 µm thick strings identified as prominent labral muscles (Fig. 2I). They attach at the base of the labrum and can be followed along its inner wall.

### Phosphatocopines

**Figure 4:**
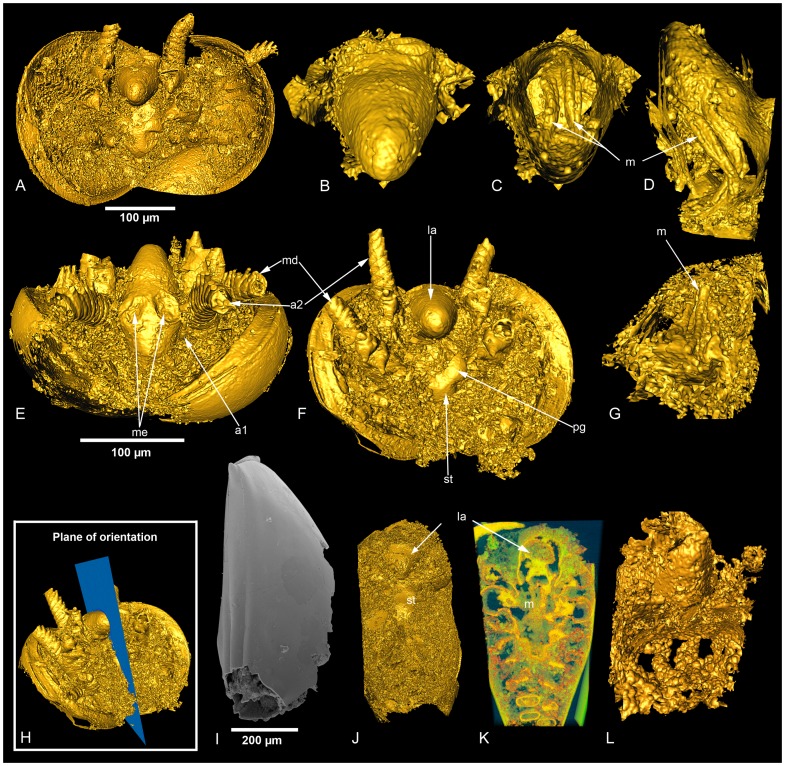
3D-rendering of SRXTM datasets and SEM image of analyzed phosphatocopines. (A) Ventral view (isosurface) of *Hesslandona* sp. (LO 11409 t) showing the internal anatomy. (B–D) Close-up (isosurface) of labrum of the same specimen, showing external surface (B) and labral muscle bundles in different views; C in same view as B, D with apex of labrum pointing upwards (see also Video S1). (E,F) Isosurface in antero-ventral (F) and ventral (G) views of *Hesslandona* sp. (LO 11410 t). (G) Close-up and cross section of labrum of that same specimen, revealing the labral muscles. Apex of labrum pointing downwards and muscles viewed from the posterior. (H) Specimen LO 11410 t showing the plane of orientation (Y-plane). (I) SEM-micrograph of *Hesslandona trituberculata* (LO 11411 t). (J) 3D-rendering of a tomographic dataset (cropped isosurface) showing the internal anatomy and labrum of the same specimen. (K) Internal virtual cross-section (cropped volrender) of the same specimen, showing numerous structures. (L) Close-up of the labrum of the same specimen, showing the labral muscles. Abbreviations: a1, first antenna; a2, second antenna; la, labrum; m, muscle; me, median eye; ps, paragnaths; st, sternum.

Phosphatocopines represent the most common and diversely represented ‘Orsten’ fossils and they are thought, like most or all of the Swedish ‘Orsten’ taxa, to have been part of the meiofauna [4]. Maas *et al*. [3] described 14 phosphatocopine species of the Cambrian Swedish ‘Orsten’ including the two taxa analysed herein. The three specimens (Fig. 4) included in this study range in size from 200 to 800 µm, measured along the hinge line. The largest one belongs to *Hesslandona trituberculata* and the two smaller specimens are assigned to *Hesslandona* sp. The labrum of the analysed phosphatocopines is a prominent bulbous, conical structure with a broad basal area protruding from the anterior part of the animal (Fig. 4A–B, E–F, J). Our analysis allowed the identification of string-like structures within the labrum that are interpreted as muscles used to control labral movement. A prominent labrum muscle pair is observed in all three of our investigated specimens. Each muscle is composed of several muscle fibers, giving them a bundle-like appearance (Fig. 4C–D, G, K–L, Video S1). They originate approximately one-third of the distance from the apex of the labrum, where they are attached to the caudal side of the labrum wall at approximately 45 degrees from the Y-plane (Fig. 4C, D, H). From the attachment point they protrude towards the mouth in an arched fashion (Fig. 4D) and appear to insert on the paragnaths in the sternum. With the exception of these structures, no internal organs or muscles could be unambiguously detected in our specimens.

## Discussion

### Taphonomy

The fossils of the ‘Orsten’ Konservat-Lagerstätte are preserved by means of phosphate encrustation and impregnation of both external and, as shown in this study, internal organs of animals during early diagenesis, producing pristine three-dimensional preservation of fossils ([4], [6] and references therein). The ‘Orsten’ type preservation can be expected to vary between different samples, but also between specimens and different structures within a single specimen. The fixation of histological preparations of extant arthropods provides an interesting analogue. The requirements for a good fixation of internal structures depend on a number of variables, a crucial one being rapid penetration of the tissues by suitable chemicals. An external cuticle delays the absorption of fixation liquids. In the case described here, the cuticle was probably partly ripped up and the animal was rapidly submerged in phosphate-rich fluids.

**Figure 5:**
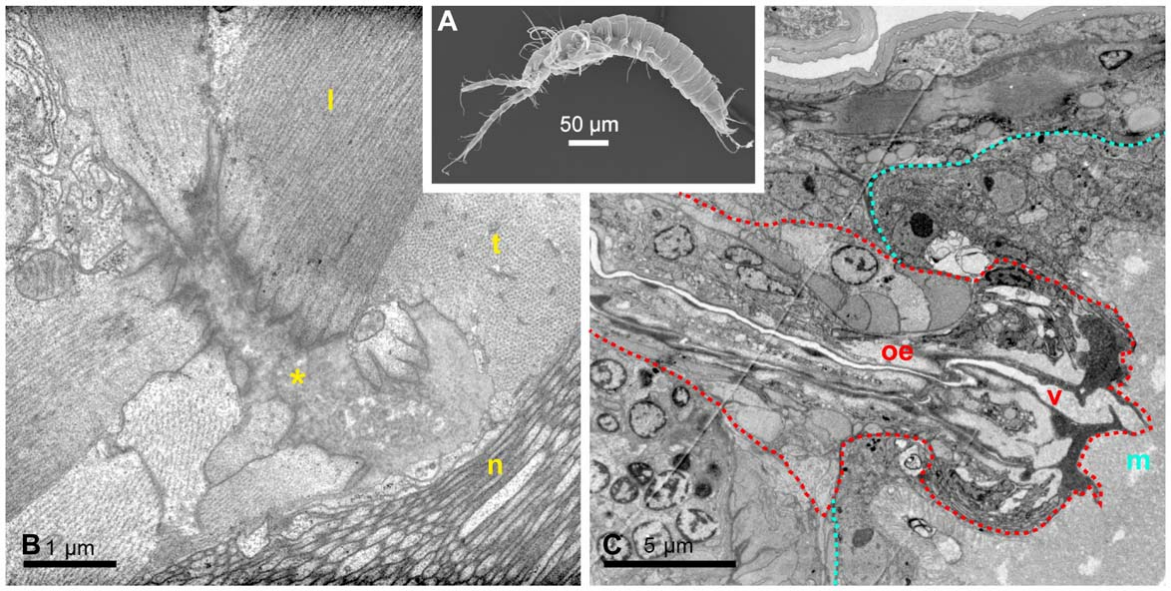
External and internal anatomy of the extant mystacocarid *Derocheilocaris typica*. (A) SEM micrograph showing the external morphology of *D. typica* in lateral view. (B) TEM micrograph showing the ventral endoskeletal-muscle plate in the mandibular region in high magnification. The asterisk (*) labels the tendinous muscle attachment. Muscle cells approach the tendon from different directions. Muscle fibers are sectioned longitudinally (l) and transversely (t). Note that the muscle fibers fill most of the muscle cells. The ventral nerve chain is labelled (n). Anterior is to the left. (C) TEM micrograph; midsagittal section through the telescoping transition between oesophagus (oe) and the midgut (m). The dashed blue line follows the contour of the midgut whereas the red dashed line follows that of the oesophagus. The oesophagus ends inside the midgut with a valve (v).

Our results indicate that some internal structures preserve fairly well whereas others deteriorate fast. Those which seem to withstand destruction best are the muscles and tendons. Their cell content of a “skeleton” of myosin and actin fibres (Fig. 5) probably contributes to the resistance to decay. In the same way, tight units of microvilli and cells may resist deterioration better. The remaining tissue is probably attached to the more resistant organs as an amorphous substance, giving them a fuzzy appearance and also contributing to a slight increase in size. The sometimes coarse, ‘bubble-like’ appearance of the internal matter could also result from diagenetic overgrowth and/or bacterially-mediated remnants of soft tissue [4].

Considering that the few specimens analysed herein contain remains of internal tissues and organs, albeit in variable detail and state of preservation, our results suggest that internal soft-tissue preservation in ‘Orsten’ fossils is more common than previously thought and demonstrate significant potential for future studies.

### Comparative anatomy and implications for mode of life

Skaracarids are maxillopod crustaceans, closely related to the extant Copepoda and Mystacocarida (Figure 5) [28], whereas phosphatocopines are regarded as the sister-group of the Eucrustacea within the Labrophora *sensu* Siveter *et al*. [29] (see also [3], [30]). The internal structures in our specimens are compared to similar relevant structures in extant crustacean relatives.

#### *Skara*

Dahl [31] investigated the topography of the crustacean head. In crustaceans filtering food particles from a current produced by the appendages, the mouth opening is ventral and directed backwards and the oesophagus makes a curve in the back of the head to meet the midgut. With other modes of feeding, exemplified as browsing and gnawing, the mouth is more anterior in position and the oesophagus is more or less straight. Dahl [31] concluded that there is a correlation between the mode of feeding and the topography of the anterior head region. He also postulated that filter-feeding was the ancestral feeding mode of crustaceans.

Müller and Walossek ([13]:26) suggested that *Skara* lived near or on the sea floor and proposed that because of its ‘cephalo-maxillipedal’ filter apparatus and lack of structures developed for biting or grasping, it was a typical filter-feeder with a preference for small-sized food such as detrital particles or bacterial films. Our results show that *Skara minuta* has a straight oesophagus and a mouth in an anterior position (and ventrally directed) and, due to the slanting anterior portion of the head, the mouth is slightly uplifted (Figs. 1,2,3). The labrum is relatively short and hardly covers the mouth. By contrast, in extant filter-feeding crustaceans the labrum is long, covering the mouth and sometimes reaching the level of the maxillipeds [19]. Another common filter-feeder feature is a broad and flat labrum forming a preoral cavity, which can be sealed on the sides [32]. The combined anatomical characteristics of *Skara minuta* thus suggest a mode of life where filter-feeding seems less probable. Rather scraping or brushing the substrate would be the primary way of feeding.

The simplest form of intestine in crustaceans, such as cephalocarids and mystacocarids (Fig. 5), consists of an oesophagus that joins the midgut without intervening structures [19], [33], a condition normally occurring in filter-feeding animals. *Skara minuta* has a similarly simple structure although in this case with a presumed alternative mode of feeding.

Müller and Walossek ([13]:22) noted that numerous external muscle scars observed on various parts of the body of *Skara* indicate a great number of muscles. However, because no internal structures were preserved, Müller and Walossek [13] found it speculative to reconstruct or interpret internal features based only on the muscle scars. In this study, we are able to address some of these issues.

Arthropods develop specific internal attachments for muscles. In extant taxa they are connective, i.e. formed by muscle tendons (Figure 5), or cuticular formed by invaginations from the cuticle (apodemes), or a combination of both. The attachment sites can detach from the cuticle and epidermis and form an internal skeleton. The endoskeletal variation is considerable within arthropod taxa [34]. The attachment sites can be both intersegmental and intrasegmental. Trunk segments are usually equipped with dorsal and ventral transverse structures for the insertion of dorsal and ventral longitudinal muscles as well as dorso-ventral and extrinsic limb muscles (e.g. [27]). More complicated endoskeletal bars and plates are found in the head region where intersegmental and intrasegmental elements coalesce. The latter are situated above and close to the ventral nerve cord.

In the *S. minuta* specimen analysed, two structures are interpreted as endoskeletal remains. Dorsally, in the trunk segments below the arthrodial membrane (Fig. 1D), transverse thickenings, or tendons (Fig. 2A, B), indicate longitudinal muscles of a size that would allow great flexibility, similar to those of the highly movable cephalocarids [27].

The head plate consists of a combination of endoskeleton and muscles. The fossil material does not allow a separation between the two. Since no apodemes were found in our specimen it is likely that the endoskeletal structures were tendon-like and thus not particularly elaborate. The muscles to the head appendages are large, especially those associated with the second antennae and mandibles. This indicates muscle strength and a good capacity to handle food in a mode described above.

#### Phosphatocopines

The labral muscle equipment has been investigated in some extant crustaceans. A highly movable labrum was described for species belonging to the conchostracan phyllopod genus *Caenesteriella* by Larink [35]. Six pairs of essentially dorso-ventral muscles line up along the long axis of the labrum. The distal swelling of the labrum contains a network of muscles. A pair of longitudinal muscles insert proximally in the ventral portion of the labrum and in the head behind the compound eyes. The dorso-ventral muscles flatten the labrum and the longitudinal muscles open the buccal cavity. Together, these two functions aid in the collection of food.

Similar functions are found in the cephalocarid crustacean *Hutchinsoniella macracantha* [32]. Two groups of dorso-ventral muscles widen the labrum and the buccal cavity. One longitudinal muscle pair spans the ventral length of the labrum and one other muscle pair, which is dorso-ventrally inserted into the ventral labral surface and into the dorsal head-shield, opens the buccal cavity. A transverse muscle pair counters the movement of the dorso-ventral muscles.

The functional pattern is repeated in the mystacocarid crustacean *Derocheilocaris remanei* [33] which has three pairs of dorso-ventral muscles inside the labrum and two pairs for operating the labrum. One pair inserts longitudinally into the middle of the dorsal surface of the labrum and into the head, and the other pair extends from the labrum to the dorsal head capsule.

The musculature of the labrum of extant crustaceans can serve as a template only in a functional context. A strict morphological pattern serving all crustacean taxa is not present.

The longitudinal muscle pair found in the phosphatocopine specimens fulfils one of the above-discussed functions, namely moving the labrum up and down, thus opening the buccal cavity. A speculative explanation for the appearance of musculature in the labrum from an evolutionary point of view is that opening of the buccal cavity could take preference over a more sophisticated armament, allowing also a flattening of the labrum. The lack of dorso-ventral muscles in the investigated phosphatocopines may imply that these muscles appeared at a later stage in the evolution of crustaceans; however, it could also simply be a preservational artefact.

## Supporting Information

Video S1 **Video clip showing the labrum of** ***Hesslandona*** **sp. (LO 11409 t).** Rotation showing the internal labral muscles.
(MP4)
